# News and Events of the Week

**Published:** 1911-04-08

**Authors:** 


### News and Events of the Week.

It is officially announced in Dublin that the Lord Lieutenant has appointed Mr. William Richard Dawson, M.D., F.R.C.P., to be Inspector of Lunatics in Ireland.

At the Central London Throat and Ear Hospital, Gray's Inn Road, W.C., on Tuesday, April 11, at 3.45 p.m., a lecture on "Anæsthetics" will be given by Dr. Kingsford.

Dr. Joseph Dawson Crawford, of Rodney Street, Liverpool, late senior surgeon to the Liverpool Hospital for Cancer and Skin Diseases, who died on February 7, aged seventy-four, left estate of the gross value of £52,762, with net personalty £7,608.

Dr. Ya Mei Kin, the first Chinese lady to obtain a medical diploma, delivered an address on the subject of "The Opportunity for Women's Education in China" at the annual general meeting of the Friends' Foreign Mission Association. Dr. Ya Mei Kin has practised medicine in China and Japan, and has lectured in America. Since her return to China a few years ago, Dr. Kin has worked against opium-smoking and foot-binding.

The library of J. B. Ballière et fils, 19 rue Haute-feuille, Paris, has just issued a new bibliography of the medical sciences in which are classified all the important volumes and memoirs which have appeared on any given subject. The volume consists of 192 pages in-8, and is published at the price of 1 franc, but the publishers will be pleased to send a copy gratis to readers of this journal on receipt of English stamps to the value of 2½d. (to cover cost of postage), and on mentioning the name of this journal.

The views of Dr. G. Arbour Stephens on "The lack of calcium salts in the food of troops on active service as a predisposing cause of enteric fever and dysentery," as set forth in an article which appeared in The Hospital of February 4 last (p. 557) have been placed before the War Office Special Commission on the subject.

At Guy's Hospital the Gull Studentship in Pathology and allied subjects is vacant. The studentship is open to candidates under thirty years of age who have studied in the Medical School of Guy's Hospital, is of the annual value of £150, and is tenable for three years. Further particulars may be obtained from the Secretary of the Board of Electors, to whom application must be addressed at the office of the Medical School, not later than April 20. The elected student will be expected to commence work on May 1.

Reporting upon the Education Committee's proposals as to the improvement of the arrangements for the medical inspection and treatment of children attending public elementary schools, the Finance Committee of the L.C.C. does not think that the Council should in any way commit itself to the establishment of a complete scheme of treatment, such as is apparently contemplated by the Board of Education, without having before it information as to at least the general lines of such scheme and an approximate estimate of its cost. It is therefore strongly of opinion that in the financial interests of the Council the Education Committee's recommendation should not be agreed to in its present form.

The following are the arrangements for next week at the Medical Graduates' College and Polyclinic, 22 Chenies Street, W.C.; Monday, April 10, at 4 p.m., Dr. Graham Little, Clinique (Skin); at 5.15 p.m., Dr. A. M. Latham, "The Treatment of Pneumonia." Tuesday, at 4 p.m., Dr. F. W. Price, Clinique (Med.); at 5.15 p.m., Mr. A. W. Mayo Robson, "The Treatment of Chronic Jaundice." Wednesday, at 4 p.m., Mr. L. H McGavin, Clinique (Surg.); at 5.15 p.m., Dr. F. W. Tunnicliffe, "The Rationale of Clothing." Thursday, at 4 p.m., Mr. James Cantlie, Clinique (Surg.); at 5.15 p.m., Dr. F. L. Golla, "Disturbances of Ocular Movements in Diseases of the Nervous System."

Professor E. H. Starling, M.D., F.R.S., has resigned his membership of the Senate of London University as representative of the Faculty of Medicine. Dr. W. P. Herringham takes the place of Dr. H. A. Caley as a representative of the Faculty of Medicine. Professor Starling was granted leave of absence until the end of the current session on account of illness. The following appointments were made: Mr. L. V. Cargill, F.R.C.S., L.R.C.P., Governor of King's College School, Wimbledon; Mr. F. T. Travers, M.B., B.S., Governor of the Maidstone Grammar Schools; Dr. E. G. Graham Little, M.D., F.R.C.P., representative of the University at the celebration of the Centenary of the Royal University of Breslau in August, 1911. The title of Emeritus Professor of Clinical Medicine at University College was conferred on Sir Thomas Barlow, M.D., F.R.S.

### KITS COTY HOUSE.

It is so seldom that anything of historical interest is offered by auction at the severely businesslike Mart in the City that the above is worth mention. This Kentish Cromlech is supposed to have been erected about 1,800 or 2,000 years b.c., and by some is mentioned as the probable burial place of Catigern, the British prince who was slain in single combat with Horsa at Aylesford a.d. 435. The monument, which is scheduled under the "Ancient Monuments Act of 1882," is about a mile and a half from Aylesford, and is to be offered early next month by Messrs. Hampton and Sons. It provides excellent sites for week-end villas.

